# Modulation of thalamocortical relay by basal ganglia in Parkinson’s disease and dystonia

**DOI:** 10.1186/1471-2202-12-S1-P275

**Published:** 2011-07-18

**Authors:** Yixin Guo, Choongseok Park, Min Rong, Robert M Worth, Leonid L Rubchinsky

**Affiliations:** 1 Department of Mathematics, Drexel University, Philadelphia, PA, 19104, USA; 2 Department of Mathematical Sciences and Center for Mathematical Biosciences, Indiana University Purdue University Indianapolis, Indianapolis, IN 46202, USA; 3 Department of Neurological Surgery, Indiana University School of Medicine, Indianapolis, IN 46202, USA; 4 Stark Neurosciences Research Institute, Indiana University School of Medicine, Indianapolis, IN 46202, USA

## 

Two major neurological disorders – Parkinson’s disease and dystonia – are believed to involve pathology in the activity of the basal ganglia, a subcortical brain structure, whose output nuclei (internal Globus Pallidus, GPi) projects to thalamus and modulates thalamocortical relay. While these disorders may ultimately involve different network and cellular pathologies, some pathological physiology may be shared between them because surgical treatment of both conditions includes surgical lesion or electrical stimulation to GPi (pallidotomy and GPi DBS). This work compares the thalamocortical relay responses to inhibitory inputs from internal segment of GPi in Parkinson’s disease and in dystonia.

Experimental data suggest that both conditions are marked by stronger oscillatory activity. In dystonia this activity becomes pathologically strong in the theta and alpha bands [1,2], while in Parkinson’s disease this is the beta-band activity [3]. The activity itself is patterned in time [4], complicating the computational study of its role. To compare the modulation of thalamocortical relay, we use experimental data recorded from GPi of human subjects with Parkinson’s disease or dystonia and study the difference of the quality of thalamocortical relay in these conditions following the computational setup, presented earlier in [5].

The results of the study of the “hybrid” system (computational model of TC cell modulated by experimental data) reveal a substantial similarity in the properties of relay in Parkinson’s disease and in dystonia. TC relay fidelity is substantially impaired due to the pathological pattern of GPi signals in both conditions. The results are robust with respect to variations of the model details and the types of incoming excitatory synaptic input.

The results suggest that even though the rhythmicity in Parkinson’s disease and dystonia are confined to different frequency bands, their effect on the dynamics of downstream circuits is similar. Thus given the differences in dystonic and parkinsonian symptoms these results suggest the existence of mechanisms beyond pathological rhythmicity and thalamocortical relay in at least in one of the conditions. On the other hand, overlap in some motor deficits of dystonia and Parkinson’s disease may be attributed to the existence of similar pathological rhythmicities and the resulting deficiencies of thalamic relay.
